# Effectiveness of unconditional cash transfers combined with lipid-based nutrient supplement and/or behavior change communication to prevent stunting among children in Pakistan: a cluster randomized controlled trial

**DOI:** 10.1093/ajcn/nqab341

**Published:** 2021-10-06

**Authors:** Sajid Bashir Soofi, Shabina Ariff, Gul Nawaz Khan, Atif Habib, Sumra Kureishy, Yasir Ihtesham, Masawar Hussain, Arjumand Rizvi, Muhammad Sajid, Naveed Akbar, Cecilia Garzon, Saskia de Pee, Zulfiqar A Bhutta

**Affiliations:** Department of Paediatrics and Child Health, Aga Khan University, Karachi, Pakistan; Centre of Excellence in Women and Child Health, Aga Khan University, Karachi, Pakistan; Department of Paediatrics and Child Health, Aga Khan University, Karachi, Pakistan; Department of Paediatrics and Child Health, Aga Khan University, Karachi, Pakistan; Department of Paediatrics and Child Health, Aga Khan University, Karachi, Pakistan; World Food Programme, Islamabad, Pakistan; World Food Programme, Islamabad, Pakistan; Centre of Excellence in Women and Child Health, Aga Khan University, Karachi, Pakistan; Centre of Excellence in Women and Child Health, Aga Khan University, Karachi, Pakistan; Centre of Excellence in Women and Child Health, Aga Khan University, Karachi, Pakistan; Benazir Income Support Programme, Government of Pakistan, Islamabad, Pakistan; World Food Programme, Islamabad, Pakistan; World Food Programme, Rome, Italy; Centre of Excellence in Women and Child Health, Aga Khan University, Karachi, Pakistan

**Keywords:** stunting, unconditional cash transfer, lipid-based nutrient supplement, social and behavior change communication, children

## Abstract

**Background:**

In Pakistan, the prevalence of stunting among children younger than 5 y has remained above WHO critical thresholds (≥30%) over the past 2 decades.

**Objectives:**

We hypothesized that an unconditional cash transfer (UCT) combined with lipid-based nutrient supplement (LNS) and/or social and behavior change communication (SBCC) will prevent stunting among children 6–23 mo of age.

**Methods:**

This was a 4-arm, community-based cluster randomized controlled trial conducted in the district of Rahim Yar Khan, Pakistan. A total of 1729 children (UCT, *n* = 434; UCT + SBCC, *n* = 433; UCT + LNS, *n* = 430; and UCT + LNS + SBCC, *n* = 432) were enrolled at 6 mo of age and measured monthly for 18 mo until the age of 24 mo.

**Results:**

At 24 mo of age, children who received UCT + LNS [rate ratio (RR): 0.85; 95% CI: 0.74, 0.97; *P* = 0.015) and UCT + LNS + SBCC (RR: 0.86; 95% CI: 0.77, 0.96; *P* = 0.007) had a significantly lower risk of being stunted compared with the UCT arm. No significant difference was noted among children who received UCT + SBCC (RR: 1.03; 95% CI: 0.91, 1.16; *P* = 0.675) in the risk of being stunted compared with the UCT arm. The pooled prevalence of stunting among children aged 6–23 mo was 41.7%, 44.8%, 38.5%, and 39.3% in UCT, UCT + SBCC, UCT + LNS, and UCT + LNS + SBCC, respectively. In pairwise comparisons, a significant impact on stunting among children in UCT + LNS (*P* = 0.029) and UCT + LNS + SBCC (*P* = <0.001) was noted compared with the UCT arm.

**Conclusions:**

UCT combined with LNS and UCT + LNS + SBCC were effective in reducing the prevalence of stunting among children aged 6–23 mo in marginalized populations. UCT + SBCC was not effective in reducing the child stunting prevalence. This trial was registered at clinicaltrials.gov as NCT03299218.

## Introduction

Child stunting is the devastating result of poor nutrition during the first 1000 d of life (1). Children with stunting have a higher risk of morbidity, mortality, short height as adults, reduced cognition, and poor school performance and earn less as adults, which contributes to an intergenerational cycle of malnutrition and poverty (2–8). Worldwide, around 149 million children younger than 5 y were stunted in 2020, and more than half of these children live in Asia (1).

In Pakistan, the child stunting prevalence has remained above WHO critical thresholds (≥30%; very high) over the past 2 decades. In 2018, stunting prevalence was 40%, highest among children living in rural areas (43%) and in the lowest wealth quintile (51%) of the national population (9). With half of the nation's population living in Punjab, the province reported one of the highest prevalences of stunting, especially among children living in the poorest households (47% in the lowest wealth quintile) (9). Once further disaggregated by divisions, the highest rate of stunting and poverty was noted in district Rahim Yar Khan, where 2 of 5 children younger than 5 y were stunted and from households in the lowest wealth quintile (9).

Cash transfers are well-recognized interventions to improve maternal and child nutrition by addressing household poverty and reducing social vulnerability. Several studies have reported that cash transfer programs appear to be an effective approach to increase the uptake of preventive health care services, improve household food consumption and dietary diversity, reduce childhood mortality, and improve child health and development outcomes, including a significant reduction in low birth weight, child stunting, and wasting (10–16).

One of the key interventions to enhance nutrition knowledge and promote healthy dietary practices is social and behavioral change communication (SBCC) (17, 18). Recent evidence on SBCC interventions has found improvements in infant and young child feeding (IYCF) practices in several developing countries (18–22), although the impact of SBCC on IYCF and child growth is mixed, depending on the study design, duration of intervention, and study setting (18–22).

A lipid-based nutrient supplement (LNS) was initially developed for the prevention of malnutrition among 6- to 23-mo-old children and treatment of moderate malnutrition among children aged 6–59 mo (23). Previous studies have shown mixed effects on the incidence of stunting and wasting, as well as the mean change in weight for height, weight gain, height for age, and height gain (24–32). These mixed results may be due to differences in targeting different age group children and nutritional status, using different types and quantities of nutritional supplements, and implementation in different settings.

Building on existing evidence, we aimed to test our hypotheses that unconditional cash transfers combined with LNS–medium quantity (LNS-MQ) and/or SBCC provided to children aged 6–24 mo, for a duration of 18 mo, could prevent stunting by 24 mo of age.

## Methods

### Study design

A community-based cluster randomized controlled trial with 4 arms was conducted in district Rahim Yar Khan, Pakistan. The district is in the southern part of Punjab province, and the total area of the district is 11,880 km^2^. The district comprises 4 tehsils and 122 union councils with a registered population of 4.8 million in 2018. The overall literacy rate is 50% (41% females and 58% males) aged 15–49 y. Around 13% of the population has access to improved sources of drinking water (piped water), 75.6% have access to improved sanitation, and 90.6% have access to electricity. Only 7.8% of households own agricultural land and 56% own livestock (33, 34). Informed consent was obtained from all parents or caregivers prior to the recruitment in the study, data collection, and anthropometric measurements. The Ethics Review Committee of Aga Khan University and the National Bioethics Committee of Pakistan have approved the study for human subject research.

### Participants

Children at 6 mo of age, living in poorest households in the lowest wealth quintile (poverty score of ≤16.17), were eligible to participate in the study. Eligible households and children were identified through the World Bank's Poverty Scorecard and Benazir Income Support Programme's (BISP's) beneficiary registry. The Poverty Scorecard, ranging from 0 to 100, was developed using a proxy mean test based on the National Socio-Economic Registry data. The data include household demographics; head of household characteristics; housing size, structure, and quality; access to and type of toilet facilities; child status; and ownership of agricultural land and livestock. Households with a poverty score of 0.00–16.17 are registered as BISP beneficiary households. Lady health worker (LHW) registers and BISP beneficiary committees were used to identify the presence of eligible children among BISP beneficiary households. Children with severe acute malnutrition and/or chronic illnesses were not enrolled in the study but were referred to the nearest health facility for treatment. Once enrolled, monthly follow-up visits were conducted for data collection (from 7 to 24 mo of age) across the study arms. Enrollment and monthly follow-ups were conducted from May 2017 to July 2019.

### Randomization and blinding

We used the existing LHW catchment area as the unit of randomization to deliver the intervention package. LHWs are working under the Integrated, Reproductive, Maternal, Newborn, Child Health & Nutrition Programme (IRMNCH&NP), funded and implemented by the Government of Punjab, Pakistan. Each LHW serves a population of 1000–1500 or ∼200 households. Before randomization, we arbitrarily chose 3 tehsils (Rahim Yar Khan, Sadiq Abad, and Khan Pur) within southern Punjab, taking distance and travel time into consideration, to ensure safe, efficient, and effective data collection by the research team. A 2-stage stratified random sampling strategy was used to minimize the risk of contamination among the study arms. In the first stage, probability proportional to size was used to select union councils with a higher probability of LHW coverage proportionate to population size (larger LHW catchment areas). To compensate for the first stage, second-stage sampling was used to ensure equal probability of selecting LHW catchment areas and identifying an equal number of eligible children per LHW catchment area or clusters. Of the 1600 LHW catchment areas or clusters identified, a total of 200 clusters were randomly selected and assigned into 1 of 4 study arms. Each study arm was assigned 50 clusters. Randomization was conducted by an independent statistician who was not involved in the study. The blinding of study participants and study arms was not possible from data collection teams and investigators as they were responsible for supervising the provision of LNS-MQ and SBCC sessions. However, data analysts remained blinded to study participants and study arms until the final data analysis was completed.

### Intervention

The intervention packages included an unconditional cash transfer (arm 1: UCT), a UCT combined with LNS (arm 2: UCT + LNS), a UCT combined with SBCC (arm 3: UCT + SBCC), and a UCT combined with SBCC and LNS (arm 4: UCT + SBCC + LNS). All 4 intervention arms received 5000 Pakistani rupees or US$32 on a quarterly basis through BISP distribution points after biometric verification.

In the UCT + LNS arm, in addition to the quarterly UCT, a locally produced LNS in medium quantity (called Wawamum) was provided to children from 6 to 24 mo of age by the data collection team. Each enrolled child received 30 sachets of LNS on a monthly basis for 18 mo from 6 to 24 mo of age. Further details on the intervention packages and nutritional content of the LNS-MQ are reported elsewhere (35).

The SBCC package was designed based on formative research conducted by the research team. The formative research was based on social cognitive theory, principles of social marketing, and an integrated behavioral model. The package was implemented to inform and promote healthy behaviors among mothers and children, especially on maternal nutrition, IYCF practices, use of LNS, and water, sanitation, and hygiene (WASH). LHWs were responsible for delivering the SBCC messages during their routine monthly household visits and quarterly community sessions using a picture booklet. LHWs were provided with an intensive 2-d training on SBCC, focused on communication skills, introduction of complementary feeding, and child dietary diversity. A 1-d annual refresher training was also provided to the LHWs during the study period. A total of 18 individual household sessions and 6 community sessions on healthy behaviors were provided to all mothers, caregivers of the enrolled children, and community members.

In the LNS + SBCC arm, the enrolled children received a daily sachet of LNS, whereas their mothers or caregivers were provided with the SBCC sessions. In the UCT + LNS + SBCC arm, enrolled children and their mothers or caregivers received the complete package of interventions.

### Procedures

We hired 6 data collection teams locally from the study district. One data collection team consisted of 2 female data collectors with a minimum educational qualification of 12 y and 1 male team leader with a minimum educational qualification of 14 y. The study teams received a 5-d hands-on training on study objectives, methodology, questionnaires, interviewing techniques, anthropometry, hemoglobin testing, and research ethics. Initially all questionnaires were developed in English and translated into Urdu and then translated back into English by an independent person. These questionnaires were pretested in the field before the data collection. The Android Studio was used to develop a data collection application and a secure storage database on MySQL Workbench. Data collection teams collected data on tablets from mothers of enrolled children from 6 to 24 mo of age. Data on sociodemographics, anthropometry, IYCF practices, child health, WASH, access to health services, existing interventions, and household food consumption practices were collected at the time of recruitment. Data on the child's length and weight, child morbidity and mortality, health-seeking behaviors, adherence to intervention, and IYCF practices were collected during monthly follow-up visits.

### Outcomes

The primary study outcome was to reduce the prevalence of stunting among children at the age of 24 mo. Secondary outcomes included reduction in the prevalence of wasting and underweight among children at 24 mo of age (34).

### Sample size calculations

A sample size of 400 children per arm was estimated based on 45% baseline stunting prevalence in district Rahim Yar Khan to detect a 20% difference (power of 0.80, α of 0.05, and intracluster correlation of 0.0008) in the prevalence of stunting among children at the age of 24 mo (34, 36). The prevalence of stunting is assumed to be in the range of ±5%. The CV was calculated using the range, whereas the intracluster correlation coefficients were derived from the CV formula (37, 38).

### Statistical analysis

Study intervention was the primary exposure, modeled as a 4-level categorical variable. The *z* scores at 6, 12, 18, and 24 mo for weight for age, length for age, and weight for length were established using WHO growth standards. Stunting, wasting, and underweight were defined as length-for-age *z* score (LAZ) of <–2 SD, weight-for-length *z* score (WLZ) of <–2 SD, and weight-for-age *z* score (WAZ) of <–2 SD, respectively.

This study was geographically clustered, prior to modeling baseline characteristics at the age of 6 mo, by analyzing differences in means and proportions among the study arms using the χ^2^ test for proportions and ANOVA for continuous variables. The multivariable analysis, performed by adjusting the effect of baseline covariates, was found to differ across arms. Mean anthropometric measurements and *z* scores were reported at 6, 12, 18, and 24 mo and compared using linear regression (34).

Adjusted prevalence and rate ratios (RRs) with corresponding 95% CIs and *P* values were obtained from a generalized linear model using a log link and binomial distribution. When the log binomial model did not converge, a Poisson distribution with link(log) was used. The estimates were reported at 6, 12, 18, and 24 mo. Analysis was adjusted for sex, BISP poverty score, and respective malnutrition status at initiation of study (i.e., 6 mo). An additional pooled regression analysis was obtained to see the combined effect of intervention groups on primary outcomes adjusted for age and sex of the child. All possible pairs of intervention were tested using the regression postestimation “test” command. All analyses accounted for the clustering effect and were performed with Stata statistical software (version 16; StataCorp LLC). Analysis was conducted by intention to treat.

## Results

### Participant characteristics

**FIGURE 1:**
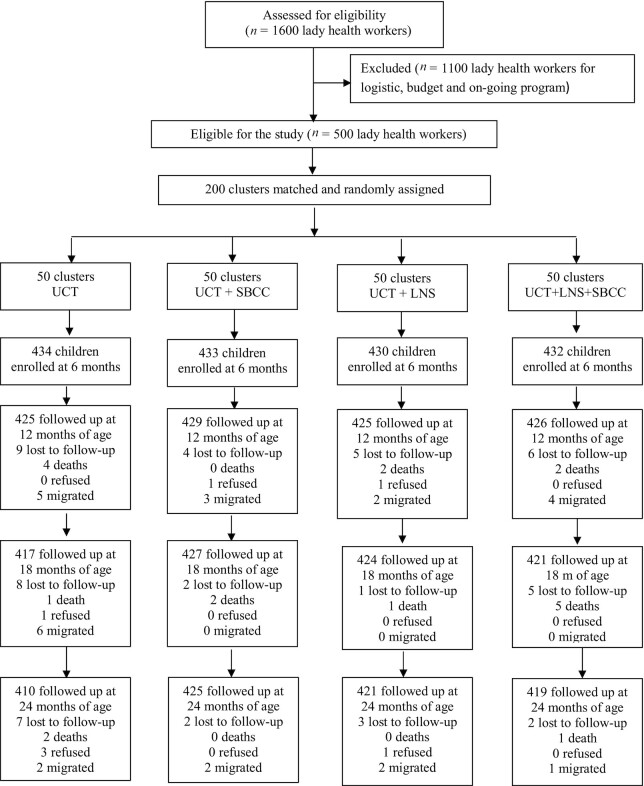
Trial profile. LNS, lipid-based nutrient supplement; SBCC, social and behavior change communication; UCT, unconditional cash transfer.

A total of 1729 children were enrolled from May 26, 2017, to January 31, 2018. There was >95% retention across the 4 study arms. Fifty-four (3.1%) children were lost to follow-up: 27 (1.5%) migrated out of the study area, 20 (1.2%) died before 24 mo of age, and 7 (0.4%) refused to participate in the study (**Figure 1**).

At baseline, all study arms had similar characteristics related to household size; access to an improved source of drinking water and sanitation; fuel for cooking; mean age of mothers; occupation of mothers; prevalence of underweight among mothers; parental education; child's sex and mean age; prevalence of stunting, wasting, and underweight; vaccination coverage; and breastfeeding rates among enrolled children (**Table 1**).

**TABLE 1:** Baseline characteristics of households, mothers, and children by study arms^1^

Variable	UCT (*n* = 434)	UCT + SBCC (*n* = 433)	UCT + LNS (*n* = 430)	UCT + SBCC + LNS (*n* = 432)
Household size	7.7 ± 2.5	7.5 ± 2.5	7.9 ± 2.5	7.7 ± 2.5
BISP poverty score	11.32 ± 0.67	12.47 ± 0.67	11.00 ± 0.66	11.55 ± 0.66
Mother age, y	28.7 ± 7.8	31.0 ± 7.8	28.6 ± 7.8	29.6 ± 7.8
Mother years of schooling	1.5 ± 3.7	0.9 ± 3.7	1.4 ± 3.7	1.0 ± 3.7
Total pregnancies	4.2 ± 2.6	4.4 ± 2.6	4.0 ± 2.6	4.2 ± 2.6
Mother BMI, kg/m^2^	22.4 ± 3.7	21.7 ± 3.8	22.0 ± 3.8	22.0 ± 3.8
Mother height, cm	154.2 ± 6.3	155.2 ± 6.4	154.1 ± 6.4	153.3 ± 6.4
Father years of schooling	2.9 ± 4.3	3.1 ± 4.3	3.0 ± 4.3	2.8 ± 4.3
Father height, cm	164.6 ± 6.4	165.0 ± 6.6	167.0 ± 6.6	167.6 ± 6.5
Child age, mo	6.2 ± 0.3	6.3 ± 0.3	6.3 ± 0.3	6.2 ± 0.3
Child length, cm	64.4 ± 2.8	64.5 ± 2.7	64.1 ± 2.7	63.9 ± 2.7
Child weight, kg	6.7 ± 1.3	6.7 ± 1.3	6.7 ± 1.3	6.7 ± 1.3
Improved water	424 (97.7)	433 (100.0)	418 (97.2)	418 (96.8)
Improved sanitation facility	313 (72.1)	254 (58.7)	323 (75.1)	175 (40.5)
Wood as fuel for cooking	355 (81.8)	270 (62.4)	278 (64.7)	360 (83.3)
Mother's occupation				
Housewife	392 (90.3)	429 (99.1)	381 (88.6)	421 (97.4)
Working woman	42 (9.7)	4 (0.9)	49 (11.4)	11 (2.5)
BMI of mother				
Underweight (<18.5)	74 (18.3)	87 (20.5)	83 (19.6)	78 (18.7)
Normal (18.5–24.9)	232 (57.3)	254 (59.9)	242 (57.2)	249 (59.7)
Overweight (25–29.9)	72 (17.8)	66 (15.6)	73 (17.3)	70 (16.8)
Obese (≥30)	27 (6.7)	17 (4.0)	25 (5.9)	20 (4.8)
Child sex				
Male	240 (55.3)	224 (51.7)	226 (52.6)	225 (52.1)
Female	194 (44.7)	209 (48.3)	204 (47.4)	207 (47.9)
Stunted	108 (24.9)	115 (26.6)	120 (27.9)	115 (26.8)
Underweight	103 (23.8)	113 (26.1)	111 (25.9)	108 (25.1)
Wasted	54 (12.5)	53 (12.2)	55 (12.8)	52 (12.1)
Ever breastfed	434 (100.0)	433 (100.0)	430 (100.0)	432 (100.0)
Breastfeeding at 6 mo	402 (92.6)	398 (91.9)	396 (92.1)	379 (87.7)
All vaccination by 6 mo	407 (93.8)	362 (83.6)	406 (94.4)	397 (91.9)
Illness during last 2 wk				
High-grade fever	337 (77.7)	307 (70.9)	240 (55.8)	225 (52.1)
Diarrhea	248 (57.1)	169 (39.0)	167 (38.8)	90 (20.8)
ARI	224 (51.6)	91 (21.0)	102 (23.7)	12 (2.8)

1 Proportions and means are accounted for clustering. Values are presented as mean ± SD or *n* (%). ARI, acute respiratory infection;; BISP, Benazir Income Support Programme; LNS, lipid-based nutrient supplement; SBCC, social and behavior change communication; UCT, unconditional cash transfer.

### Child growth status

**Table 2** shows the growth trends in children at 6, 12, 18, and 24 mo of age, including anthropometric measurements such as length, weight, WAZ, LAZ, and WLZ. Pairwise tests indicated that there was no significant difference in length at any point between the study arms, except in the UCT + SBCC arm compared with the UCT + LNS + SBCC arm at 6 mo of age (*P* = 0.015). At 24 mo of age, the mean weight was found to be significantly different between the UCT arm and the UCT + SBCC arm (*P* = 0.025), the UCT arm and the UCT + LNS + SBCC arm (*P* = 0.001), and the UCT + LNS arm and the UCT + LNS + SBCC arm (*P* = 0.039). Similarly, mean WAZ at 24 mo had a significant difference between the UCT arm and the UCT + SBCC arm (*P* = 0.046), the UCT arm and the UCT + LNS + SBCC arm (*P* = 0.001), and the UCT + LNS arm and the UCT + LNS + SBCC arm (*P* = 0.031). There were no significant differences observed in the mean LAZ between the study arms at 6, 12, 18, and 24 mo of age. At 24 mo of age, a significant difference was noted in the mean WLZ between the UCT arm and the UCT + SBCC arm (*P* = 0.013), the UCT arm and the UCT + LNS arm (*P* = 0.040), the UCT arm and the UCT + LNS + SBCC arm (*P* = <0.001), and the UCT + LNS arm and the UCT + LNS + SBCC arm (*P* = 0.024).

**TABLE 2:** Anthropometric measurements in children at 6, 12, 18, and 24 mo of age^1^

	Mean ± SD (95% CI)				*P* values (pairwise comparison)^2^					
Variables	UCT (A)	UCT + SBCC (B)	UCT + LNS (C)	UCT + LNS + SBCC (D)	A vs. B	A vs. C	A vs. D	B vs. C	B vs. D	C vs. D
Length, cm										
6 mo	64.37 ± 2.74 (64.07, 64.67)	64.55 ± 2.73 (64.18, 64.92)	64.12 ± 2.74 (63.84, 64.40)	63.94 ± 2.74 (63.65, 64.23)	0.417	0.183	0.054	0.093	0.015	0.382
12 mo	70.73 ± 2.93 (70.39, 71.08)	70.32 ± 2.91 (69.96, 70.68)	70.70 ± 2.99 (70.39, 71.01)	70.59 ± 3.04 (70.26, 70.92)	0.094	0.866	0.584	0.163	0.307	0.675
18 mo	75.95 ± 3.40 (75.64, 76.27)	75.79 ± 3.46 (75.42, 76.17)	76.12 ± 3.48 (75.78, 76.47)	75.98 ± 3.49 (75.64, 76.33)	0.527	0.483	0.896	0.273	0.470	0.597
24 mo	80.68 ± 3.50 (80.38, 80.97)	80.59 ± 3.55 (80.18, 81.00)	80.95 ± 3.59 (80.62, 81.28)	80.89 ± 3.54 (80.56, 81.22)	0.731	0.234	0.381	0.220	0.285	0.812
Weight, kg										
6 mo	6.73 ± 1.27 (6.59, 6.86)	6.71 ± 1.26 (6.62, 6.81)	6.73 ± 1.27 (6.62, 6.84)	6.65 ± 1.27 (6.53, 6.76)	0.863	0.974	0.317	0.845	0.353	0.343
12 mo	8.35 ± 1.37 (8.21, 8.49)	8.22 ± 1.36 (8.10, 8.34)	8.29 ± 1.40 (8.16, 8.41)	8.13 ± 1.42 (8.02, 8.24)	0.114	0.494	0.009	0.469	0.250	0.046
18 mo	9.30 ± 1.14 (9.16, 9.45)	9.34 ± 1.16 (9.21, 9.47)	9.29 ± 1.17 (9.16, 9.42)	9.20 ± 1.17 (9.07, 9.34)	0.731	0.887	0.260	0.609	0.143	0.336
24 mo	10.51 ± 1.39 (10.36, 10.65)	10.26 ± 1.41 (10.12, 10.41)	10.35 ± 1.43 (10.21, 10.48)	10.16 ± 1.41 (10.04, 10.29)	0.025	0.172	0.001	0.388	0.294	0.039
Weight-for-age *z* score										
6 mo	–1.28 ± 1.62 (–1.43, –1.12)	–1.33 ± 1.62 (–1.45, –1.21)	–1.26 ± 1.61 (–1.40, –1.13)	–1.35 ± 1.61 (–1.50, –1.20)	0.521	0.894	0.440	0.473	0.817	0.442
12 mo	–1.06 ± 1.43 (–1.19, –0.93)	–1.14 ± 1.42 (–1.26, –1.02)	–1.08 ± 1.46 (–1.21, –0.96)	–1.21 ± 1.48 (–1.33, –1.09)	0.260	0.747	0.073	0.471	0.408	0.120
18 mo	–1.23 ± 1.10 (–1.36, –1.10)	–1.16 ± 1.12 (–1.28, –1.04)	–1.21 ± 1.13 (–1.32, –1.09)	–1.29 ± 1.13 (–1.41, –1.16)	0.415	0.787	0.495	0.580	0.125	0.325
24 mo	–1.10 ± 1.17 (–1.21, –0.98)	–1.27 ± 1.19 (–1.38, –1.15)	–1.19 ± 1.21 (–1.30, –1.09)	–1.35 ± 1.19 (–1.46, –1.25)	0.046	0.267	0.001	0.369	0.262	0.031
Length-for-age *z* score										
6 mo	–1.21 ± 1.14 (–1.32, –1.10)	–1.21 ± 1.14 (–1.35, –1.06)	–1.33 ± 1.13 (–1.46, –1.20)	–1.34 ± 1.13 (–1.45, –1.23)	0.983	0.095	0.118	0.257	0.153	0.902
12 mo	–1.69 ± 1.13 (–1.80, –1.57)	–1.84 ± 1.12 (–1.97, –1.70)	–1.67 ± 1.15 (–1.78, –1.55)	–1.67 ± 1.17 (–1.79, –1.55)	0.091	0.806	0.860	0.084	0.082	0.989
18 mo	–2.01 ± 1.16 (–2.12, –1.90)	–2.04 ± 1.18 (–2.17, –1.92)	–1.90 ± 1.18 (–2.01, –1.79)	–1.96 ± 1.19 (–2.07, –1.84)	0.692	0.145	0.517	0.144	0.310	0.525
24 mo	–1.97 ± 1.07 (–2.07, –1.88)	–1.96 ± 1.09 (–2.08, –1.83)	–1.85 ± 1.10 (–1.94, –1.75)	–1.87 ± 1.08 (–1.97, –1.77)	0.854	0.053	0.159	0.219	0.291	0.778
Weight-for-length *z* score										
6 mo	–0.61 ± 1.64 (–0.79, –0.42)	–0.65 ± 1.64 (–0.77, –0.53)	–0.48 ± 1.64 (–0.60, –0.35)	–0.55 ± 1.64 (–0.69, –0.40)	0.636	0.149	0.507	0.024	0.279	0.444
12 mo	–0.23 ± 1.37 (–0.37, –0.10)	–0.27 ± 1.36 (–0.38, –0.16)	–0.30 ± 1.40 (–0.43, –0.16)	–0.49 ± 1.42 (–0.64, –0.34)	0.680	0.534	0.002	0.701	0.018	0.047
18 mo	–0.35 ± 1.08 (–0.49, –0.22)	–0.25 ± 1.10 (–0.37, –0.13)	–0.39 ± 1.11 (–0.52, –0.25)	–0.46 ± 1.11 (–0.62, –0.31)	0.235	0.754	0.246	0.135	0.035	0.424
24 mo	–0.10 ± 1.15 (–0.22, 0.03)	–0.34 ± 1.17 (–0.48, –0.21)	–0.31 ± 1.18 (–0.44, –0.18)	–0.51 ± 1.16 (–0.65, –0.38)	0.013	0.040	<0.001	0.715	0.103	0.024

1 Estimates are accounted for clustering. LNS, lipid-based nutrient supplement; SBCC, social and behavior change communication; UCT, unconditional cash transfer.

2 Linear regression was used for comparison of means by study arms. Pairwise comparison was done between study arms.

### Primary outcome

At 24 mo of age, children enrolled in the UCT + LNS arm (RR, 0.85; 95% CI: 0.74, 0.97; *P* = 0.015) and the UCT + LNS + SBCC arm (RR, 0.86; 95% CI: 0.77, 0.96; *P* = 0.007) had a significantly lower risk of being stunted compared with the UCT-only arm. There was no significant difference noted among children enrolled in the UCT + SBCC arm (RR, 1.03; 95% CI: 0.91, 1.16; *P* = 0.675). Stunting prevalence at 24 mo of age was found to be 48.5% (95% CI: 44.8, 52.2) in the UCT-only arm, 49.8% (95% CI: 45.2, 54.3) in the UCT + SBCC arm, 41.1% (95% CI: 36.4, 45.9) in the UCT + LNS arm, and 41.6% (95% CI: 37.8, 45.4) in the UCT + LNS + SBCC arm. When compared with the prevalence of stunting in the UCT arm at 24 mo of age, a 15% difference in the prevalence of stunting was found in the UCT + LNS arm and 14% in the UCT + LNS + SBCC arm (**Table 3**).

**TABLE 3:** Adjusted prevalence and rate ratio of stunting, wasting, and underweight in children at 6, 12, 18, and 24 mo of age^1^

		Stunting			Wasting			Underweight		
Age and study arms	*n*	Prevalence	Rate ratio	*P* value	Prevalence	Rate ratio	*P* value	Prevalence	Rate ratio	*P* value
6 mo										
UCT	430	24.4 (20.3, 28.5)			12.7 (8.6, 16.8)			23.7 (19.5, 28)		
UCT + SBCC	429	26.3 (22.0, 30.6)			12.0 (8.8, 15.2)			26.0 (21.8, 30.2)		
UCT + LNS	430	26.8 (21.8, 31.7)			12.8 (9.5, 16.0)			25.8 (21.9, 29.8)		
UCT + LNS + SBCC	430	26.2 (22.1, 30.4)			12.1 (9.0, 15.2)			25.0 (20.2, 29.8)		
12 mo										
UCT	382	39.6 (35.2, 44.0)	Reference		10.0 (7.0, 12.9)	Reference		19.4 (15.8, 22.9)	Reference	
UCT + SBCC	376	41.5 (37.4, 45.6)	1.05 (0.89, 1.23)	0.563	10.8 (7.6, 13.9)	1.08 (0.71, 1.63)	0.716	20.9 (17.3, 24.4)	1.08 (0.85, 1.37)	0.528
UCT + LNS	398	35.5 (30.8, 40.2)	0.90 (0.75, 1.07)	0.235	8.4 (5.6, 11.3)	0.85 (0.57, 1.25)	0.405	16.5 (12.6, 20.5)	0.85 (0.63, 1.16)	0.307
UCT + LNS + SBCC	412	35.6 (32.3, 38.8)	0.90 (0.78, 1.04)	0.151	8.9 (6.2, 11.5)	0.89 (0.59, 1.35)	0.589	20.4 (17.2, 23.6)	1.06 (0.84, 1.33)	0.642
18 mo										
UCT	385	51.1 (47.1, 55.1)	Reference		8.9 (5.7, 12.0)	Reference		23.4 (19.4, 27.4)	Reference	
UCT + SBCC	400	51.5 (47.1, 55.9)	1.01 (0.90, 1.13)	0.897	9.3 (6.8, 11.9)	1.05 (0.67, 1.65)	0.829	22.1 (19.0, 25.3)	0.94 (0.74, 1.2)	0.644
UCT + LNS	405	42.6 (38.1, 47.1)	0.83 (0.72, 0.96)	0.013	7.5 (4.8, 10.3)	0.85 (0.48, 1.49)	0.563	20.4 (16.4, 24.4)	0.87 (0.67, 1.14)	0.315
UCT + LNS + SBCC	408	47.0 (42.8, 51.2)	0.92 (0.83, 1.02)	0.120	7.1 (4.4, 9.9)	0.8 (0.45, 1.41)	0.440	21.6 (18.0, 25.2)	0.92 (0.75, 1.13)	0.435
24 mo										
UCT	395	48.5 (44.8, 52.2)	Reference		7.9 (5.5, 10.3)	Reference		20.9 (17.4, 24.4)	Reference	
UCT + SBCC	406	49.8 (45.2, 54.3)	1.03 (0.91, 1.16)	0.675	8.3 (5.6, 11.0)	1.04 (0.67, 1.63)	0.857	22.5 (18.6, 26.4)	1.08 (0.85, 1.36)	0.539
UCT + LNS	417	41.1 (36.4, 45.9)	0.85 (0.74, 0.97)	0.015	5.5 (2.9, 8.1)	0.70 (0.39, 1.24)	0.217	19.4 (15.4, 23.3)	0.93 (0.71, 1.22)	0.578
UCT + LNS + SBCC	404	41.6 (37.8, 45.4)	0.86 (0.77, 0.96)	0.007	6.3 (4.1, 8.5)	0.79 (0.51, 1.23)	0.298	21.4 (18.1, 24.6)	1.02 (0.83, 1.25)	0.832

1 Values are presented as prevalence or rate ratio (95% CI), with *P* values obtained from a generalized linear model using a log link and binomial distribution. When the log binomial model did not converge, a Poisson distribution with link(log) was used. Rate ratio and prevalence were adjusted by sex, Benazir Income Support Programme poverty score, and baseline nutritional status, and standard errors were accounted for clusters. LNS, lipid-based nutrient supplement; SBCC, social and behavior change communication; UCT, unconditional cash transfer.

### Secondary outcomes

At the age of 24 mo, no significant difference in the risk of being wasted was noted between the study arms. Wasting prevalence at 24 mo of age was 7.9% (95% CI: 5.5, 10.3) in the UCT-only arm, 8.3% (95% CI: 5.6, 11.0) in the UCT + SBCC arm, 5.5% (95% CI: 2.9, 8.1) in the UCT + LNS arm, and 6.3% (95% CI: 4.1, 8.5) in the UCT + LNS + SBCC arm. When compared with the prevalence of wasting in the UCT arm at 24 mo of age, a 30% difference in the prevalence of wasting was found in the UCT + LNS arm, whereas a 20% difference was found in the UCT + LNS + SBCC arm (Table 3).

At 24 mo of age, no significant difference in the risk of being underweight was found in the study arms compared with the UCT-only arm. The prevalence of underweight at 24 mo of age was 20.9% (95% CI: 17.4, 24.4) in the UCT-only arm, 22.5% (95% CI: 18.6, 26.4) in the UCT + SBCC arm, 19.4% (95% CI: 15.4, 23.3) in the UCT + LNS arm, and 21.4% (95% CI: 18.1, 24.6) in the UCT + LNS + SBCC arm (Table 3).

### Pooled study results

The pooled prevalence rate of stunting during 6–24 mo of age was 41.7%, 44.8%, 38.5%, and 39.3% for the UCT-only, UCT + SBCC, UCT + LNS, and UCT + LNS + SBCC arms, respectively. When compared with the UCT-only arm, the UCT + LNS and UCT + LNS + SBCC arms were found to have significantly reduced prevalence of stunting (*P* = 0.029 and *P* < 0.001). No significant differences were noted in any other study arms (**Table 4**).

**TABLE 4:** Pooled and adjusted prevalence of stunting, wasting, and underweight in children 6–24 mo of age^1^

Variable	Stunting	Wasting	Underweight
UCT	41.7 (37.9, 45.4)	9.5 (7.6, 11.3)	21.9 (18.7, 25.2)
UCT + SBCC	44.8 (40.3, 49.3)	9.7 (7.8, 11.6)	22.1 (18.5, 25.8)
UCT + LNS	38.5 (34.3, 42.7)	8.4 (6.5, 10.3)	20.8 (17.3, 24.3)
UCT + LNS + SBCC	39.3 (35.1, 43.4)	8.6 (6.5, 10.7)	21.6 (17.8, 25.4)
*P* values (pairwise comparison)			
UCT vs. UCT + SBCC	0.147	0.858	0.727
UCT vs. UCT + LNS	0.029	0.231	0.529
UCT vs. UCT + LNS + SBCC	<0.001	0.608	0.597
UCT + SBCC vs. UCT + LNS	0.415	0.151	0.823
UCT + SBCC vs. UCT + LNS + SBCC	0.107	0.452	0.883
UCT + LNS vs. UCT + LNS + SBCC	0.562	0.424	0.910

1 Values are presented as prevalence (95% CI) and *P* values. Prevalence is accounted for cluster, sex, and age. *P* values were obtained from a generalized linear model using a log link and binomial distribution. LNS, lipid-based nutrient supplement; SBCC, social and behavior change communication; UCT, unconditional cash transfer.

The pooled prevalence rate of wasting during 6–24 mo of age was found to be 9.5%, 9.7%, 8.4%, and 8.6% for the UCT-only, UCT + SBCC, UCT + LNS, and UCT + LNS + SBCC arms, respectively. Compared with the UCT only arm, there was no significant difference noted in the pooled prevalence of wasting among all other study arms.

The pooled prevalence rate of underweight during 6–24 mo of age was 21.9%, 22.1%, 20.8%, and 21.6% for the UCT-only, UCT + SBCC, UCT + LNS, and UCT + LNS + SBCC arms, respectively. Compared with the UCT-only arm, there was no significant difference found in the pooled prevalence of underweight among all other study arms (Table 4).

### Adherence to intervention

In the UCT + LNS arm, the reported adherence to LNS among children during the entire duration of the study was 82.7 ± 11.2%. Similarly, the reported adherence to LNS among children in UCT + LNS + SBCC arm was 94 ± 11.3%. Compared with the UCT + LNS arm, a significant difference (82.7% compared with 94.1%; *P* = <0.001) was noted in the reported compliance to LNS among children in the UCT + LNS + SBCC arm (**Table 5**).

**TABLE 5:** Compliance of LNS among children 6–24 mo of age^1^

Variable	UCT + LNS (*n* = 428), Mean ± SD	UCT + LNS + SBCC (*n* = 431), Mean ± SD	*P* value
Days observed	533.5 ± 41.7	529.7 ± 41.9	0.282
Days LNS received	500.5 ± 60.9	509.9 ± 61.1	0.024
Days LNS consumed	418.2 ± 83.1	481.4 ± 83.4	<0.001
Percent compliance to LNS (days consumed/days observed * 100)	82.7 ± 11.2	94.1 ± 11.3	<0.001
Number of sachets received	474.9 ± 87.2	496.9 ± 87.5	<0.001
Number of sachets consumed	382.6 ± 84.6	419.2 ± 84.9	<0.001
Number of LNS sachets shared with other family members	50.3 ± 34.5	15.3 ± 34.6	<0.001

1 Estimates are accounted for clustering and based on maternal recall. LNS, lipid-based nutrient supplement; SBCC, social and behavior change communication; UCT, unconditional cash transfer.

Based on the mothers’ recall, 6.2%, 86.5%, 70.8%, and 93.5% of mothers in the UCT arm, UCT + SBCC arm, UCT + LNS arm, and UCT + LNS + SBCC arm reported receiving messages on health and nutrition education during the study on a monthly basis, respectively. Around 38% of mothers in the UCT + SBCC arm and 52% in the UCT + SBCC + LNS arm reported that they received messages on complementary feeding for their children. Maternal recall on the importance of dietary diversity for their child was low in the UCT + LNS arm (32.6%), whereas in the UCT + SBCC arm (94.7%), most mothers were able to recall these messages. Similarly, most mothers with children enrolled in study arms with SBCC recalled receiving messages on maternal nutrition, WASH, and benefits of LNS (**Table 6**).

**TABLE 6:** Percentage of SBCC messages provided to mothers of children aged 6–24 mo^1^

Variable	UCT (*n* = 434)	UCT + SBCC (*n* = 433)	UCT + LNS (*n* = 430)	UCT + LNS + SBCC (*n* = 432)
Received any health education session/messages on monthly during 6–24 mo of age?				
Yes	6.2	86.5	70.8	93.5
No	93.9	13.5	29.2	6.5
Received health session/messages on				
Introduction of complementary feeding	11.3	37.8	6	52
Dietary diversity for the child	63.2	94.7	32.6	39.1
Continuation of BF for 2 y	29.5	74.1	20.2	54.2
Benefits of LNS	0	0.2	93.5	86
Not sharing of LNS with others	0	0.1	72.7	75.3
Maternal nutrition/balance diet	19.1	80.4	26	57
WASH	29.5	91.9	68.2	56.9
Other messages	17.2	0.6	0.5	0.1

1 Estimates are accounted for clustering and based on maternal recall. BF, breastfeeding;; LNS, lipid-based nutrient supplement; SBCC, social and behavior change communication; UCT, unconditional cash transfer; WASH, WASH, water, sanitation, and hygiene.

On average, 4600 PKR were transferred to each BISP beneficiary household with an enrolled child on a quarterly basis during the study period. In the UCT + SBCC + LNS arm, the cash was used on food items (2606 ± 1569 PKR), followed by transport (1020 ± 1041 PKR), health and medicine (316 ± 1185 PKR), clothing (20 ± 326 PKR), and school fees (11 ± 79 PKR). Similar spending trends were noted in other study arms (**Table 7**).

**TABLE 7:** Household utilization of unconditional cash transfers^1^

Variable	UCT (*n* = 430)	UCT + SBCC (*n* = 431)	UCT + LNS (*n* = 426)	UCT + LNS + SBCC (*n* = 430)
UCT received quarterly (PKR)	4703 ± 267	4694 ± 267	4540 ± 266	4560 ± 267
Spending on food	2434 ± 1569	2067 ± 1571	2696 ± 1562	2606 ± 1569
Spending on transport	891 ± 1041	656 ± 1042	1190 ± 1036	1020 ± 1041
Spending on health/medicine	796 ± 1185	577 ± 1187	314 ± 1180	316 ± 1185
Spending on clothing	62 ± 326	41 ± 327	48 ± 325	20 ± 326
Spending on education/school fee	15 ± 79	57 ± 79	16 ± 79	11 ± 79
Spending on other items	506 ± 948	1296 ± 949	275 ± 944	587 ± 948

1 Values are presented as mean ± SD. Estimates are accounted for clustering and based on maternal recall. LNS, lipid-based nutrient supplement; SBCC, social and behavior change communication; UCT, unconditional cash transfer.

## Discussion

This study was designed to estimate the effect of UCT in combination with LNS, SBCC, and LNS + SBCC on the reduction of risk in stunting among children at 24 mo of age. The SBCC sessions and cash transfers were implemented through the existing health and social protection systems of the Government of Pakistan. At 24 mo of age, a 15% reduction in the prevalence of stunting was found in the UCT + LNS (41.1%; 95% CI: 36.4%, 45.9%) and 14% in the UCT + LNS + SBCC (41.6%; 95% CI: 37.8%, 45.4%) arms compared with the UCT arm (48.5%; 95% CI: 44.8%, 52.2%). A similar reduction in the prevalence of stunting has been found in previous studies (39–41). No significant difference was noted in the prevalence of wasting and underweight among children in all study arms compared with the UCT arm at 12, 18, and 24 mo of age. The prevalence of stunting increased with age between 6 and 24 mo of age across all study arms; however, the increase was at a smaller increment in the UCT + LNS and UCT + LNS + SBCC arms.

When compared with the UCT arm, UCT + SBCC was not an effective approach to reduce the prevalence of stunting among children at 24 mo of age living in households from the lowest wealth quintile. These findings are similar to other studies from low- and middle-income countries (42–44). Several explanations are possible for the lack of impact of SBCC on stunting, including the short 18 mo of exposure time for knowledge acquisition on IYCF and changing of optimal behaviors and practices (45).

A significantly higher rate of compliance and a low rate of sharing LNS in the UCT + LNS + SBCC arm were observed compared with the UCT + LNS arm. The higher compliance rate and limited sharing of LNS in the UCT + LNS + SBCC arm can be attributed to SBCC sessions, especially messages on the importance of LNS, delivered by the LHWs.

One of the major strengths of this study was the use of an existing health system and a national social protection program to implement the intervention packages. This existing integrated infrastructure allowed for capacity building of the local LHWs, lady health supervisors (LHSs), mothers, and caregivers. It also created a strong sense of ownership by the Government of Pakistan. Additional strengthens are a high rate of compliance to LNS and low loss to follow-up. This study also adds to the existing evidence on the feasibility and effectiveness of community-based programs to integrate health, nutrition, and social protection to improve child nutritional outcomes.

There was some inevitable limitation in the study design. First, this study could not be blinded due to visible intervention packages. However, data collection was standardized, questionnaires were structured, and data collection teams were rotated between the study arms in order to limit any bias. Second, most UCT beneficiaries were not the mothers of the enrolled children but rather the grandmother or another household member. This limited the ability of mothers to be decision makers and spend the UCT on their child's basic and dietary needs.

In conclusion, the use of UCT combined with LNS and SBCC was shown to be effective in reducing the prevalence of stunting in children at 24 mo of age in low- and middle-income settings. Scaling up of the UCT, in combination with LNS and SBCC sessions, is recommended to improve the nutritional status of children living in marginalized populations. Further larger-scale trials are needed to confirm these findings and to determine the sustainability and long-term impact of these intervention packages on child undernutrition.

## Data Availability

Data described in the manuscript, code book, and analytic code will be made available upon request from the corresponding author.

## Notes

Funding was provided by the Federal Ministry for Economic Cooperation and Development (BMZ) through the World Food Programme, Pakistan (PAK/2016/038).

Abbreviations used: BISP, Benazir Income Support Programme; IRMNCH&NP, Integrated, Reproductive, Maternal, Newborn, Child Health & Nutrition Programme; IYCF, infant and young child feeding; LAZ, LAZ, length-for-age *z* score; LHS, lady health supervisor; LHW, lady health worker; LNS, lipid-based nutrient supplement; LNS-MQ, lipid-based nutrient supplement–medium quantity; RR, rate ratio; SBCC, social and behavior change communication; UCT, unconditional cash transfer; WASH, water, sanitation, and hygiene; WAZ, weight-for-age *z* score; WLZ, weight-for-length *z* score.
